# The British Red Cross in North-West Europe

**Published:** 1946

**Authors:** K. M. Agnew

**Affiliations:** Deputy Secretary, Council of British Societies for Relief Abroad


### THE BRITISH RED CROSS IN NORTH-WEST EUROPE

BY

LIEUTENANT-COLONEL K. M. AGNEW,
D.S.O., M.C., R.A. (retired).

*Deputy Secretary, Council of British Societies for Relief Abroad.*

An address delivered at the Annual Meeting of the Bristol Branch of the British Red Cross Society on Tuesday, May 28th, 1946.

---

### ORGANISATION

This is an account of the organisation and work of the British Red Cross in France, Germany and Holland, from the beginning of the invasion of Europe in 1944. The Commission is divided into three main parts :

(*a*) The Military Welfare Unit.
(*b*) The Foreign Relations Section.
(*c*) The Civilian Relief Unit.

### MILITARY WELFARE UNIT

The *Military Welfare unit* carries out the traditional work of the Red Cross and Order of St. John in the care and welfare of sick and wounded of the fighting forces during war. In the arrangement for sending food-parcels and other comforts to prisoners of war and the staffing of Convalescent Homes, Relatives' Hostels, etc., this unit works intimately with the army and under the general directions of the Director of Medical Services of the army with which it is functioning.

*Hospital Supplies.*—The unit had been got together in London during the winter of 1943-44, and was held in readiness, complete with men and equipment, for the coming invasion of the " fortress of Europe." Comforts and hospital necessities, based on experience gained in other theatres of war, had been accumulated and lay packed ready for immediate shipment, together with motor transport and camp equipment. The personnel had been chosen as carefully as conditions allowed in those difficult days. " D " Day came, but not till thirty days later was the spearhead of this Expeditionary Force Unit released to land on the beaches of Normandy. They speedily installed themselves and set about their work of bringing comfort and relief to the wounded. The heart of this unit, as of similar units working on other battlefields, was the " Stores." Under huge canvas tents an amazing assortment of goods had been brought together, ranging from razor-blades to nurses' gowns, and from electric irons and dart-boards to sewing-machines ; to say nothing of other trifles such as easy chairs, pyjamas, wireless sets and tea-urns—in fact, anything that was likely to be required to make conditions happier and more comfortable for patients in their tented wards. The majority of the hospitals had been pitched in fields that lined the Bayeux-Litry road, which soon became known as Harley Street. The aim and object of the unit was to jump in and fill the gap in any emergency. To do this it had to be prepared to meet the unforeseen and to deal with the unpredictable. Hence the need for stocks of such a diverse nature, the selection of which called for a certain amount of imagination. The first demand received was from a blood-transfusion unit for blankets to cover blood-donors. Thereafter a fleet of vans and lorries were speeding along the shell-pitted roads and muddy lanes of Normandy, carrying their loads to every hospital and casualty clearing station of the B.L.A. Back-rests were one of the items supplied in their hundreds for patients suffering from chest wounds. Close on the heels of the spearhead followed the main body, which brought the unit's strength up to a very modest number in view of the work they had to do.

*Welfare Officers.*—The first batch of Welfare Officers now began to arrive. Posted in pairs to each of the general hospitals, they lived under the same conditions as the sisters, under canvas, sleeping on cots sunk below ground level to protect them from shell-bursts. Conditions were not very pleasant in the waterlogged Normandy orchards in which they lived and had their being, but these girls were invariably full of cheer. It was this quality of cheerfulness under all conditions that was perhaps their outstanding trait, and made them so deservedly popular with patients. It is not always easy to be cheerful in the small hours of the morning when you have been dragged out of bed to serve tea and cigarettes to a newly-arrived convoy of patients waiting on their stretchers in the draughty reception tent to be distributed to their respective wards, but somehow these girls always managed to do it, and, what is more, to carry on as usual next day. The duty of these welfare officers is to render to patients the hundred and one little services for which the busy ward-sisters have not the time—write their letters, bring them books and distribute cigarettes, and supply deficiencies in their kit. As often as not a man is brought off the battlefield with nothing, and he has to be supplied with soap, razor toothbrush and comb, and with something to read and to smoke. He will probably wish to write a letter to his people, and note-paper and pencil are supplied. He may be worried about his wife or children, and a cable will be sent to our London headquarters to make the necessary enquiries. Or he may just be bored, and the gramophone or a game of dominoes no longer appeal, which brings us to :—

*Diversional Therapy.*—It has long been known that the state of mind of a sick man may help or hinder his cure, and that even a broken leg will heal more quickly where anxiety is absent. But it is only of late years that this fact has been given the attention it deserves. It is this field of mental healing that the Red Cross has made particularly its own, through the agency of its welfare officers and supplies. In the early part of this war the Red Cross popularized, through the medium of its 250-odd Convalescent Homes that had been established in England, what is technically known as Diversional Therapy, or in simpler language handicrafts : particularly those adapted to the use of bed-patients—tapestry, embroidery, knitting, rug-making, etc., all of which proved very popular amongst men, many of whom displayed amazing aptitude in these feminine arts. Men who had never so much as sewn on a button in their lives made beautiful embroidery. The patient's hands thus employed, he was relieved of boredom and had less time to worry about himself. Many patients displayed excessive ardour and accomplished their work too quickly, having in mind the difficulty of obtaining fresh materials—" Look, Sister ! I've finished it ! "—proudly displaying a bit of work that should have kept him quiet for a week, completed in a day.

*Motor Ambulance Convoys.*—The Red Cross has been associated with the use of motor-ambulances for the conveyance of wounded from very early days. The first M.A.C.s in France during the 1914-18 war were given and manned by the Red Cross, and it was right and proper that they should be again represented in the late war. The first Motor Ambulance Convoy arrived in Normandy in August, 1944, manned entirely by women, with the exception of their Commanding officer. They did such excellent work that at the request of the army medical authorities two more convoys were sent out as soon as they could be made ready. These also were manned by women drivers. Thus by the spring of 1945 three Motor Ambulance Convoys were operating with the B.L.A., comprising 111 ambulances and patient-carrying buses, manned by 166 women drivers. During their combined existences these ambulances covered no less than 1,400,000 miles, and carried more than 166,000 patients. The drivers of these convoys gave an outstanding example of women doing men's work. By so doing, they liberated a corresponding number of men drivers for work in areas such as the Far East, for which women drivers were considered unsuitable. The army officers under whose command these convoys operated were never tired of singing the praises of the work done by these girl drivers. They were ready for duty by day or night, rain or shine, winter or summer, with never a complaint. And what is more, the standard at which they maintained their vehicles was unapproached by any other army unit—96 per cent., which was the record of one unit, is very close to perfection.

Then came the great trek of 400 miles from Bayeux to Brussels in September, 1944. New headquarters were established and great warehouses taken to house our stores. As the enemy retired, branches were thrown out into Holland and Germany to care for advanced hospital units, and thus maintained the Stores slogan of immediate delivery. In wartime, when a hospital needs something, it is usually needed at once, and the Stores Department had always before them the old motto "he gives twice who gives quickly." Eventually the area served extended from Bayeux to Berlin, and from Marseilles to Kiel.

*Chiropody.*—One of the chief attributes of the Red Cross is its elasticity. This permits it to try out new ideas, many of which have proved their usefulness. A comparatively recent case in point is the Mobile Chiropody Units that were equipped and supplied by the Scottish branch of the Red Cross. The first trial unit, consisting of a well-equipped motor-van and its staff of four trained women, proved such an enormous success that the army asked that two more be provided. The first unit, in the months from December, 1944, to September, 1945, treated over 7,500 patients and travelled over 8,000 miles. The other two units arrived just as the war ended, and their careers were consequently comparatively short, but they were very useful whilst they lasted.

*Convalescent Homes.*—Five Convalescent Homes were opened by the Red Cross, four in Belgium and one in Berlin, where officers and other ranks, both men and women, were received for an average stay of about ten to fourteen days. A high standard of comfort was achieved and it was the endeavour of their staffs to run them as "homes" in fact as well as in name. How well they succeeded is shown by the remark of one young Guardsman that the "place made him feel homesick." Two of these homes were especially designed for the use of army sisters in need of recuperation and rest from their exacting labours. It was but right that they who were entrusted with the care of our wounded should themselves be cared for when ill.

*Relatives.*—Another Red Cross service carried out in collaboration with the army enabled relatives of dangerously wounded men to visit them. Whenever it was thought by the hospital authorities that a patient was likely to benefit by such a visit, the relatives received a wire from the War Office, on receipt of which they were able to travel free of charge to London, where they were taken over by the Red Cross, furnished with a guide and flown to Brussels. Here again they were taken charge of by a special Red Cross service and rushed to the hospital where their relative lay. During their stay they were housed in Hostels established for this sole purpose, of which eventually there were four—two in Belgium at Brussels and Ostend, and two in Germany at Hamburg and Vlotho. It is surprising how many cases which had been regarded as hopeless "turned the corner " and recovered as a direct result of a visit from a wife or mother.

*Canteen.*—In conjunction with the Ostend Hostel, a Canteen was opened at the Dockside Railway Station in a large hall that had formerly been the station buffet. Here the wounded lay on their stretchers, reading, smoking and listening to the gramophone whilst waiting to be taken on board the hospital-carrier. From the opening day in November, 1944 to the end of August, 1945, over 18,000 casualties had passed through this canteen.

*Prisoners of War.*—The release of British prisoners of war in Germany furnished a dramatic episode in the life of this Commission. Within a couple of days the trickle of released men passing through Brussels had swollen to a river in spate. They began to arrive in their thousands by train, by road and by air. Teams of welfare officers and women secretaries were hurriedly organized and put in charge of stalls that were set up in barracks, airfields and in our own headquarters, laden with Red Cross comforts ranging from combs to towels, and razor-blades to pyjamas, not forgetting rope-soled slippers for the footsore. The released men, as they filed past the stalls, were handed the articles of kit that they were lacking, with a packet of cigarettes and a bar of chocolate for good measure. To replace these issues, twenty and more Dakota planes were arriving daily from the U.K. loaded with Red Cross comforts, and sixty-five additional tons arrived by the sea route. These activities extended far beyond the Brussels area to care for released men in forward transit camps. In all some 73,000 released men passed through Brussels, few of whom missed paying a call at the Red Cross depots.

I should like now to quote some interesting figures about this side of the work.

SERVICES RENDERED .. .. *11th July*, 1944 *to* 30*th April*, 1946.

Motor ambulance Convoys—Three :

| | |
|---|---|
| Ambulances .. .. .. .. .. | 111 |
| Miles covered .. .. .. .. .. | 1,406,800 |
| Patients carried .. .. .. .. .. | 166,195 |
| Women drivers .. .. .. .. .. | 166 |

Convalescent Homes—Four :

| | |
|---|---|
| Patients received—male and female, all ranks | 1,960 |

| | |
|---|---|
| Welfare Officers attached to Military Hospitals (peak) | 106 |
| Stores Personnel (peak) .. .. .. .. | 55 |

Stores :

| | |
|---|---|
| Value of stores issued to Military Hospitals, Allied Red Crosses, etc. .. .. | £500,000 approx. |

Motor Transport :

| | |
|---|---|
| Lorries and vans .. .. .. .. | 137 |
| Miles covered .. .. .. .. .. | 1,040,000 |

Released Prisoners of War :

| | |
|---|---|
| Provided with comforts by Brussels H.Q. office | 73,000 |

Brussels. *8th May*, 1946.

| | |
|---|---|
| Total Red Cross and St. John personnel now in the Commission .. .. .. .. | about 65 |

(*i.e.* when all Brussels H.Q., etc., are finally closed down).

### FOREIGN RELATIONS SECTION.

The work of the Foreign Relations Section is closely integrated with every other part of the Commission. Not only is it engaged in searching for missing people in hospitals, devastated areas and transit camps, but it is trying to trace and bring together families that have been separated by years of war. It also helps with the work connected with the care of British subjects in liberated countries and in Germany. It successfully helps to form friendly relationships with our sister Red Cross Organizations of America, Belgium, Denmark, France, Holland, Sweden, Switzerland, Iceland, Poland, etc., and also keeps in close liaison with the International Red Cross in Geneva. Its work is of the utmost value to the Commissioner and Deputy Commissioners in every branch of the civilian relief work that they have to carry out.

### CIVILIAN RELIEF UNIT.

*France.*—Early in 1945 a scheme was started for issuing food parcels and clothes to British-born citizens in France, but it was not until September that a comprehensive plan covering the whole of France was inaugurated.

The scheme is directed and the actual relief for the Paris Area conducted from the headquarters at 1, Avenue de Marigny, Paris. This area contains the largest number of British citizens. It is estimated that some 6,000 are now in receipt of relief in the inner and outer areas of Paris : the inner comprised the city itself and its suburbs ; the outer, the huge area surrounding the Seine Department. The figures for the issues made in the last quarter of 1945 were:

| | Inner Zone | Outer Zone |
|---|---|---|
| Food parcels .. .. .. .. | 30,544 | 4,439 |
| Clothing parcels .. .. .. .. | 4,038 | 318 |
| Blankets .. .. .. .. .. | 4,467 | 976 |

The remainder of France is divided into eight areas, corresponding in nearly all cases to the British Consulate Areas. Some of these areas are enormous; the Bordeaux area, for example, comprises sixteen Departments. The centres from which the teams work are: Bordeaux, Dinard, Lille, Lyon, Marseilles, Nice, Rouen, and Strasbourg.

The main stores of parcels are at Marseilles, Paris, and Bordeaux, with a reserve supply at Antwerp. From these stores each area is supplied by heavy lorries, and dumps are made in various places as required for the particular area. The actual distribution is made by women Welfare Drivers from Britain, Canada and South Africa. The teams vary in size from eight to two according to the size of the area and the number of British citizens in it. They work in close touch with the consuls, in consultation with whom everything is done. Without the help which the consuls give practically nothing could be done.

The Teams often are out for several weeks on end, and during these periods rely for help on the French civil and military authorities: that help is given them fully, and they are always accorded a most friendly welcome by personnel of those authorities. They find the French civilians just the same, a kindly greeting and an ever-ready help. Helped by consuls, the Red Cross has tried to *find* Britons who need its help. Information that hard-pressed people are living in some isolated spot is followed up, a visit made, and then welfare drivers call with parcels.

Everywhere they go these welfare workers make contact with the French Red Cross, other charitable organizations, the French authorities of all kinds, and the French people. Always they receive from these various people a friendliness that is a grand token of the friendship that the French feel for the British, and which the average Briton desires so much to reciprocate. All this makes a very real contribution to that true friendship between our two countries which is so essential to the well-being of Western Europe.

The Relief Organization made its last issues by March 31st, giving out enough then to cover everyone up to April 30th. In addition, each consul was left with a supply of parcels according to his requirements, so that he could continue to deal with specially needy cases after the general scheme was finished. In this they will be helped by about forty B.R.C. members who will remain with them throughout this next autumn and winter.

By April it was calculated that some 170,000 parcels had been issued throughout France. That means nearly 3,000,000 articles of food distributed in six lean months—meats, butter, margarine, rice, flour, cheese, jam, tea, sugar and chocolate. In addition to all this, in co-operation with the French Red Cross, French Salvation Army, and *Sécours Quaker*, about 150,000 food parcels have been issued to

French children suffering from malnutrition, and a considerable quantity of clothing, etc., to French of all kinds in hospitals and convalescent homes. It is hoped to continue this for several months to come.

Finally, at the end of April, as a result of a report on conditions of child welfare in St. Nazaire, in co-operation with the French and Swiss Red Cross, 500 food parcels, a selection of baby-linen and children's clothing and odd supplementary items such as soap and cod-liver-oil, were taken down by road from Paris and distributed amongst twenty schools in St. Nazaire.

*Belgium, Holland and Germany.*—I come now to the part of Civilian Relief with which I have been intimately connected as Deputy Commissioner for the past sixteen months. Before I describe in detail the work we have done I should say a few words about the organization of this particular unit.

In the early days of the war there was set up the Council of British Societies for Relief Abroad. This Council, C.B.S.R.A. as it came to be known, included, as well as the British Red Cross Society and Society of St. John of Jerusalem, nine other voluntary societies, *viz.*, Friends' Ambulance Unit, Friends' Relief Service, Scouts, Guides, Salvation Army, Catholic Committee for Relief Abroad, Jewish Committee for Relief Abroad, Save the Children Fund, and International Voluntary Service for Peace. Since the army wished to deal with only one organization, all these voluntary societies agreed to serve under the British Red Cross " umbrella."

*France and Belgium.*—In September, 1944, three Relief Sections, two from F.A.U. and one from Scouts, and one B.R.C. Hospital Section, landed in Normandy and started work helping and caring for the civilian population in the devastated areas. I should mention here that each Relief Section consisted at first of twelve men, and later of twelve men or women ; and each Hospital Section of twenty-four members all told, of whom two were medical officers, four nurses, eight nursing assistants and the remainder quartermaster, orderlies, drivers, etc. From the very start the hospital sections were composed of both men and women.

This small force did extremely good work in those early days and earned high praise from the military authorities. The Commander of the Second Army said later : " If I had known their work at the time, I should have asked for more and landed them earlier." As the armies advanced they were followed up by this small body of men and women, and when I arrived at Brussels on New Year's Eve, 1944, I found them distributed in Belgium and Holland.

Soon after my arrival five more relief sections landed at Ostend and went straight to Antwerp to help the Belgian Civil Defence workers. Antwerp at that time had become the target of all the V.1's and V.2's that the Germans could bring to bear on it. In

February six more relief sections, two hospital sections, and two laboratory sections landed at Ostend and sixteen bath and ten laundry sections, the gift of Lever Bros., arrived at Boulogne. All these sections moved up to places in Belgium and South Holland where they were most needed, caring for the civilians who by now were beginning to stream back from the battle areas as the armies fought their way into Germany.

In April, I moved my H.Q. from Brussels to Tilburg, to be ready to move into North Holland as soon as it was liberated. Then on 15th April, Belsen Concentration Camp was uncovered by the army, and in response to an urgent call from the medical authorities for assistance there we despatched six relief sections from the Antwerp area to that place of horror.

They arrived there on 21st April and at once started to help, the male members of the sections going into Horror Camp No. 1 and assisting the army to evacuate those who could be moved, and the women members improvising and supervising emergency hospitals for those who were brought out suffering from starvation, typhus, dysentery, etc.; and dying, in the hospitals alone, at the rate of thirty or forty a night. The work of our women under the most appalling conditions is an epic which should never be forgotten in the annals of the British Red Cross. Of the six sections that went there, five were B.R.C. and St. John and one F.R.S.

About that time it became apparent that U.N.R.R.A., whose responsibility it was to look after the Displaced Persons (amounting to about 1,700,000 persons in the British Zone), would not have enough teams in the field to compete with their task and at the urgent request of Civil Affairs of the Army, twelve more relief sections and four more hospital sections were asked for and in due course arrived. Early in May we started moving into North Holland, and when the Germans capitulated we at once sent relief sections into the big cities of Utrecht, Rotterdam, Amsterdam and The Hague to help the starving Dutch and to assist the Netherlands Red Cross and the Dutch doctors, nurses and welfare workers in their task of rehabilitation and resettlement.

A double hospital section was set up in South Rotterdam in the dock area and looked after 250 patients suffering from typhoid. Another hospital section provided by the Guides was opened to compete with another typhoid epidemic in Gorinchem, and others went to Delft (S.C.F.) and Maastricht (C.C.R.A.). My H.Q. went to Utrecht and eventually to The Hague.

*Germany.*—Meanwhile the remainder of the Relief Sections, namely the original eight (all male) and No. 1 B.R.C. Hospital Section, were following up the victorious armies and starting their work with the displaced persons in Germany; sorting them out, assembling them in camps, nursing the sick and generally helping the army and

U.N.R.R.A. in the terrific task of looking after one-and-a-half million ex-slave labourers and concentration-camp victims suddenly released from German domination.

We finished our work in North Holland at the end of June and with the exception of the three hospital sections still competing with typhoid and the bath and laundry sections, which were handed over to the Dutch Red Cross, we all assembled in Germany, my H.Q. being at Vlotho in Westphalia province. Welfare work in the Displaced Persons Assembly Centres continued until the end of 1945, by which time more than half the displaced persons had been repatriated and U.N.R.R.A. had produced a considerable number of teams. This enabled us to agree to the suggestion of the Army and Control Commission in Germany that some of our Relief Sections should be withdrawn from the Displaced Persons' Assembly Centres and go to the assistance of the German Welfare Societies and Red Cross. The former had, to a certain extent, survived the Nazi régime and were able to start helping Germans in need of assistance. The German Red Cross, however, under Hitler, had become completely Nazified and it was some months after the capitulation before it was allowed to start again.

Our work for the Germans consists in helping these welfare societies in a large number of ways. Some of these activities are :

1. Supplementary feeding of children between the ages of three and twelve in the Ruhr, Hamburg and other cities. In this we are assisted by the International and Swedish Red Crosses, working under our supervision.

2. Evacuation of childen from Berlin to the country for the winter 1945-46.

3. Evacuation of orphan children from Berlin to foster-parents in Westphalia.

4. Help to German Red Cross in looking after refugees from the Russian zone and Czechoslovakia.

5. Similar help to German Red Cross in meeting the 1½ million persons expelled from Poland, arranging transit camps and detraining and entraining stations.

6. Encouraging the Germans to form Youth Clubs, Discussion Groups, and generally trying to show them the British democratic way of life.

7. Welfare work with ex-concentration-camp victims and inspecting local German prisons.

8. Assisting the German welfare societies in starting and running summer camps for the children in the bombed-out cities.

I will now give you some figures to show the number of sections and personnel engaged in this Civilian Relief. When I started work in Brussels on 1st January, 1945, we had three Relief Sections and one Hospital Section in the field, a total of sixty personnel. Just before we left Holland in June, 1945, there were working there and in Germany seven Hospital Sections, thirty-six Relief Sections, two Laboratory Sections, sixteen Bath Sections, ten Laundry Sections, three Canteen Sections, two Kitchen Sections, and three Information Sections, which together with my own H.Q. amounted to nearly 1,000 personnel.

As I have already told you, we left behind in Holland the Bath and Laundry Sections, but as the work in Germany developed, an appeal for twenty-four more relief sections was made by the army and these have all now gone overseas.

When I left Germany three weeks ago, two of the seven Hospital Sections, two Laboratory Sections and three Information Sections had been disbanded, as they were no longer required, and the final totals on that date, May 5th, were :

| | Teams. | Personnel. |
|---|---|---|
| **GERMAN WELFARE WORK.** | | |
| Relief Sections .. .. .. .. | 30* | 321 |
| Kitchen /Canteen Sections .. .. | 2 | 15 |
| Transport Sections .. .. .. | 1 | 9 |
| **WORK IN DISPLACED PERSONS' CAMPS.** | | |
| Hospital Sections .. .. .. | 5 | 124 |
| Relief Sections .. .. .. .. | 26* | 253 |
| **HEADQUARTERS.** | | |
| H.Q. Staff .. } Liaison Officers, etc. } .. .. .. | — | 53 |
| Rest House Staff .. .. .. .. | — | 3 |
| Nutrition Unit .. .. .. .. | — | 4 |
| **SWEDISH RED CROSS TEAM.** | | |
| Relief Sections .. .. .. .. | 6 | } 112 |
| Transport Sections .. .. .. | 1 | |
| H.Q. Staff .. .. .. .. | — | |
| **INTERNATIONAL RED CROSS.** | | |
| Relief Sections .. .. .. .. | 7† | †30 |
| Total .. .. | | 924 |

That is a very brief description of the work of the past two years under General Lindsay in the North-West Europe Commission.

* Two Relief Sections working partly on Displaced Persons, partly on German Welfare Work.

† (These are approximate figures only).

That is the material side of it. What of the humanity side ? What a vast amount of misery, hunger, discontent and fear it represents ! Think of the French at Caen and its neighbourhood in the early days, think of the starving Dutch in North Holland, of the millions of displaced persons released from places like Belsen and Sandbostel, of those Jews, Balts, Poles and Yugo-Slavs who have no home to return to or who dare not return to their homes through fear. Finally, picture the defeated Germans themselves, suffering hunger, expulsion from their homes, and the general frustration due to the calamity of defeat which they brought on themselves.

Our reward has been gratitude shown in hundreds of different ways. I will give you some examples. A man in the canteen on Folkestone railway-station who insisted on buying me a cup of tea and a bun because he saw my Red Cross uniform and told me that as a prisoner of war in Germany he would have starved to death if he had not received Red Cross food parcels. We have seen the overwhelming joy of those whom our Foreign Relations Department have been able to join together after years of separation. The Poles in the camps beseech our welfare workers to go back to Poland with them and help to look after them in the new life they are going to take up in the greatly changed country they are returning to. The Germans themselves are deeply grateful for the way we are trying to give them a new way of life and new hope for the future.

Finally, may I say this ? During the past sixteen months I have worked with men and women of all ages, of varying political ideals, of different religious beliefs and different upbringings, and we have all worked in perfect harmony ; and the reason for that harmony was that we were working for humanity, in response to those words spoken nearly 2,000 years ago : " Go, and do thou likewise." No matter who he is, why he is suffering, whose fault it is, he is " my neighbour " and it is my duty to go and help. And it is the duty of the Red Cross, which is the only one of our societies that has a Government Charter in most countries, to take the lead amongst all the other voluntary societies in this work, which is absolutely essential to the future of the world.

We, in our small way, have been working amongst people of almost every European country, and are surely doing something towards building up an understanding between ourselves and at the same time helping them to understand each other. This can become a great force for spiritual good that might do a great deal to bring peace and goodwill amongst those with whom we are working. If we can do this, then they may carry something of the true British spirit of tolerance, kindness and good-humour back with them to their respective countries, and thus help to restore peace and goodwill to this war-stricken world.

